# Assessment of Ventilation and Perfusion in Patients with COVID-19 Discloses Unique Information of Pulmonary Function to a Clinician: Case Reports of V/P SPECT

**DOI:** 10.1177/11795484211030159

**Published:** 2021-07-20

**Authors:** Marika Bajc, Fredrik Hedeer, Ari Lindqvist, Elin Trägårdh

**Affiliations:** 1 Department of Clinical Physiology and Nuclear Medicine, Skåne University Hospital Lund, 22185, Lund, Sweden; 2 Research Unit of Pulmonary Diseases, Clinical Research Institute HUCH Ltd., Helsinki University Hospital HUS and Helsinki University, Helsinki, Finland; 3 Department of Clinical Physiology and Nuclear Medicine, Skåne University Hospital Malmö, Lund University, Lund, Sweden

**Keywords:** Ventilation, perfusion, SPECT, SARS-CoV-2, pulmonary embolism, COVID-19

## Abstract

V/P SPECT from 4 consecutive patients with COVID-19 suggests that ventilation and perfusion images may be applied to diagnose or exclude pulmonary embolism, verify nonsegmental diversion of perfusion from the ventilated areas (dead space ventilation) that may represent inflammation of the pulmonary vasculature, detect the reversed mismatch of poor ventilation and better preserved perfusion (shunt perfusion) in bilateral pulmonary inflammation and indicate redistribution of lung perfusion (antigravitational hyperperfusion) due to cardiac congestion. V/P mismatch and reversed mismatch may be extensive enough to diminish dramatically preserved matching ventilation/perfusion and to induce severe hypoxemia in COVID-19.

## Introduction

Corona virus SARS-CoV-2 may cause an extended bilateral pneumonia early in COVID-19. COVID-19 may be presented simultaneously as hyper-inflammation^1^ and immunosuppression.^2^ A subset of patients can develop a severe acute lung injury, hyper-permeability in the pulmonary microvasculature and acute respiratory distress syndrome (ARDS).^3^ A systemic coagulopathy increases the risk for pulmonary embolism (PE).^4^ COVID-19 may cause multi-organ failure affecting, for example the heart.^5^

We emphasise the clinical usefulness of functional imaging by V/P SPECT in COVID-19 to explain patient’s symptoms and to verify pathophysiological changes in the lungs and the central circulation.

## Methods

Suspected pulmonary embolism (PE) was the indication for ventilation/perfusion single-photon emission computed tomography (V/P SPECT) in 4 consecutive COVID-19 patients who sought emergency care and gave their written informed consent for the case report. COVID-19 was diagnosed from the nasopharyngeal swab with a PCR test. Imaging started with the ventilation SPECT after inhaling 99mTc-labelled solid graphite hydrophobic particles (Technegas), immediately followed by the perfusion SPECT after i.v. injection of 99mTc-labelled human albumin macroaggregates. Findings were interpreted according to EANM Guideline 2019.^6^ Ventilation/perfusion defects were quantified by calculating the percentage of total lung volume (TLV) exhibiting preserved matching ventilation/perfusion, that is total preserved lung function (TPLF in %).^6^

## Results

Table 1 presents the development of the disease from onset of symptoms and the V/P SPECT, X-ray and CT findings from the patient cases with mild symptoms to the patient cases with more severe symptoms caused by an extensive COVID-19 lung inflammation.

**Table 1.** Development of the disease from onset of symptoms and summary of V/P SPECT, X-ray and CT findings.

Clinical status, co-morbidity/condition and therapy													V/P SPECT		X-ray and CT	
Patient	Age	Initial symptoms	Progressive symptoms	SaO2%	Oxygen support	Heart rate (/min)	Respiratory rate (/min)	CRP	Blood cell counts	Liver status	Co-morbidity/condition	Therapy	V/P SPECT diagnoses	TPLF in V/P SPECT (%)	Chest X-ray at hospital admission	CTPA or CT
1	30	Cough, sniffing, myalgia, mild fever (day 9)	Dyspnoea worse (day 9)	93−95 (day 9)	None	NA	30 (day 9)	11 (day 9)	Normal (day 9)	Normal (day 9)	Pregnancy, second trimester	Home therapy, prophylactic subcutaneous LMWH (day 9)	PE, bilateral lung inflammation worse in right lung, slight diversion of perfusion from apex of right lung (day 13)	65 (day 13)	Pneumonia on the right lower lobe (day 9)	None
2	30	Sore throat, fatigue, cough, dyspnoea while sitting (day 5)	Dyspnoea worse (day 5)	95 (day 5)	None	130 (day 5)	NA	33 (day 5)	Normal (day 5)	Normal (day 5)	Pregnancy, third trimester	Hospitalisation for 5 days, prophylactic LMWH (day 5)	No PE, bilateral lung inflammation, more prominently in right lung, diversion of perfusion from basal area in right lung (day 7)	60 (day 7)	Sparse peripheral parenchymal changes basally in right lung. Discrete sparse changes in hilus on left side (day 5)	None
3	50	Cough, fever, dyspnoea, myalgia, headache and diarrhoea (day 11)	Dyspnoea worse and fever > 39°C (day 11)	88 (day 19)	Optiflow^®^ (day 19)	NA	26 (day 19)	12 (day 11)	Normal (day 11)	Mild elevation of liver enzymes (day 11)	NA	Hospitalisation for 13 days, prophylactic LMWH (day 11)	No PE, bilateral lung inflammation (in larger area than in CT), diversion of perfusion from ventilated areas in right lung (day 19)	35 (day 19)	NA	No PE in CTPA, infiltration bilaterally in posterior part of lower lobes and lateral part in left upper lobe or lingula (day 11); V/P SPECT/CT (day 19)
4	55	Cough, fever up to 39.3°C, dyspnoea (day 2)	Dyspnoea worse and fever > 39°C worse (day 2)	88 (PaCO2 7,0 kPa), PaO2 9.8 kPa) (day 2)	Optiflow^®^ (day 2)	irregular 140 (day 2)	30 (day 2)	65 (day 2)	Normal (day 2)	Mild elevation of liver enzymes (day 2)	Sarcoidosis, peripheral edema and left heart hypertrophy	Hospitalisation for 15 days, prophylactic LMWH (day 2)	No PE, extensive bilateral lung inflammation (in larger area than in CT), diversion of perfusion from ventilated areas in right lung, anti-gravitational perfusion redistribution to anterior area (hyperperfusion) as in cardiac congestion (day 3)	30 (day 3)	NA	Bilateral parenchymal infiltration in lung CT (day 3)

Optiflow^®^, oxygen therapy with a high flow nasal cannula; CRP, C-reactive protein; LMWH, low molecular weight heparin or analogue; TPLF (%), quantification of ventilation/perfusion defect and remaining lung function by calculating how many percents of the total lung volume preserved matching ventilation and perfusion;^6^ CTPA, CT pulmonary angiography; NA, not applicable.

Time interval to worsening of symptoms/hospital visits/hospital examinations from the onset of symptoms (days in parenthesis).

### Findings in cases 1 and 2

Cases 1 and 2 were pregnant women aged 30 years and in the second and third trimester of pregnancy. Their symptoms of COVID-19 started with cough, sore throat, sniffing, myalgia, fatigue, mild fever and dyspnoea. Within 5 to 9 days their condition deteriorated and they were admitted to the emergency room. Their arterial oxygen saturation (SaO2) was 93% to 95% without need of extra oxygen. Heart rate and respiratory rate were significantly elevated. The C-reactive protein (CRP) was mildly elevated supporting viral infection. Differential blood cell counts (WBC diff) and liver function tests (LFTs) remained in the normal range. Chest X-ray suggested right lower lobe pneumonia in case 1 and sparse peripheral parenchymal changes basally in the right lung and in the hilus on the left side in case 2. A prophylactic anticoagulant therapy with a low molecular weight heparin (LMWH) was administered.

In case 1, V/P SPECT was performed 4 days later. The finding confirmed PE and a bilateral lung inflammation affecting more ventilation than perfusion and being more extensive in the right lung. A minor diversion of perfusion from apex of the right lung was noticed (non-segmental V/P mismatch not typical for PE). Total preserved lung function was estimated to be 65 %. The patient was treated at home.

In case 2, V/P SPECT was done on the second day after hospitalisation. It showed no sign of PE but a bilateral reversed V/P mismatch, that is better preserved perfusion in areas with absent/reduced ventilation, typical for lung inflammation, more extensive in the right lung. In addition, a diversion of perfusion from ventilated area was seen in the right lung (non-segmental V/P mismatch not typical for PE). Total preserved lung function was estimated to be 60%. The patient was hospitalised for 5 days.

### Findings in cases 3 and 4

Cases 3 and 4 were women aged 50 and 55 years, respectively. Case 4 had known sarcoidosis and at the time of the COVID-19, peripheral edema and left heart hypertrophy in echocardiography as a co-morbidity. Their COVID-19 started with cough, fever and dyspnoea and case 3 had also myalgia, headache and diarrhoea. In case 3, elevation of temperature >39°C and dyspnoea made the patient gradually seek the emergency room after 11 days. In case 4, the temperature was >39°C from the beginning and dyspnoea was progressing rapidly making the patient seek the emergency room on the second day after onset of symptoms.

**Figure 1.**
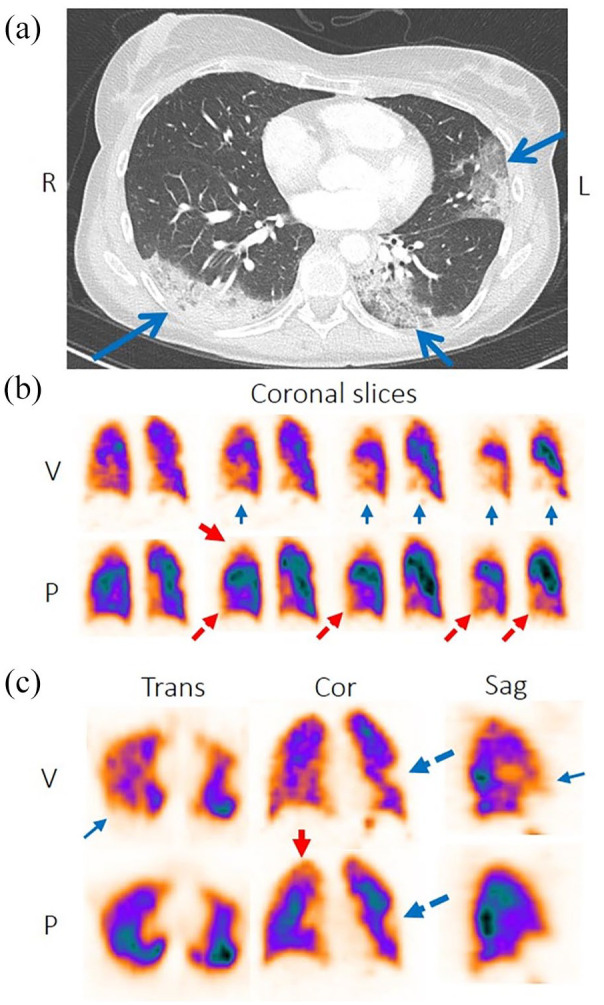
(a) CT show bilateral posterior ground-glass opacities and consolidation in the upper left lung lobe (lingula, blue arrows), (b) and (c) transversal, coronal and sagittal slices of ventilation (upper row) and perfusion (lower row). Ventilation is unevenly distributed in both lungs with areas of reduced/absent ventilation (blue arrows), more prominent on the right side and posterior lower lobe on the left side. A well delineated matched V/P defect is observed corresponding to CT consolidation change in the upper left lung lobe (lingula, dotted blue arrow). Perfusion is better preserved in areas of reduced/absent ventilation (reversed mismatch, dotted red arrows). Importantly, a diversion of perfusion from the apical part is observed (red arrow), of non-segmental character and not typical for PE, more on the right side.

In case 3, CT pulmonary angiography (CTPA) showed no signs of PE but a bilateral infiltration in the posterior part of the lower lobes and in the lateral part of the left upper lobe or lingula (Figure 1a). CRP was 12, WBC diff normal and LFTs mildly elevated. Prophylactic LMWH was administered and the patient was hospitalised. But the symptoms were progressing. Eight days later respiratory rate increased to 26/minutes, SaO2 was 88% and the patient needed oxygen therapy with a high flow nasal cannula (Optiflow^®^). V/P SPECT combined with CT presented a reversed V/P mismatch bilaterally, consistent with a lung inflammation, more extensive on the right side. The functional defect of the reversed mismatch extended to 40 % of TLV and was more extended than the inflammatory changes seen on CT (Figure 1b and c). V/P SPECT showed also a matched V/P defect in the left lung estimated to 10% of TLV that corresponded to CT changes in the lingula. In addition, a diversion of perfusion from ventilated areas was seen in the right lung extending to 15% of TLV. The total preserved lung function was estimated to be 35% (Figure 1b and c).

In case 4, the patient was hospitalised with a respiratory rate at 30/minutes, irregular tachycardia at 140/minutes, a low arterial oxygen tension (PaO2 9.8 kPa) and a retention of arterial blood carbon dioxide (PaCO2 7.0 kPa). SaO2 was 88% and the patient needed extra oxygen via Optiflow^®^. CRP was 65, WBC diff normal and LFTs mildly elevated. Prophylactic LMWH was administered. On the next day, V/P SPECT with CT showed an extensive bilateral lung inflammation. V/P SPECT showed that the inflammation was more widespread than on CT and reversed V/P mismatch extended to 45% of TLV. It was more prominent in the right lung. A diversion of perfusion from ventilated areas was observed, extending to 10% of TLV. Furthermore, anti-gravitational redistribution of perfusion to anterior areas was observed, extending to 15% of TLV, consistent with cardiac congestion. The total preserved lung function was estimated to be 30% by V/P SPECT. The treatment of the patient in the hospital continued for 15 days.

## Discussion

A diversion of perfusion from a ventilated area was the unexpected pattern observed in the V/P SPECT of all 4 patients with a lung inflammation caused by SARS-CoV-2 virus (Table 1). This V/P mismatch was not of segmental character and did not represent PE.^6^ Parenchymal inflammation causes a ventilation defect that physiologically induces vasoconstriction. Physiological vasoconstriction does not explain the diversion of perfusion which occurs in ventilated areas of the lung. In the absence of PE, angiopathy (inflammation of the pulmonary microvasculature or endothelial injury) or hyperflow bypassing alveolar perfusion remains a possible explanation of diversion of perfusion from ventilated noninjured lung areas. Pulmonary vessels in patients with COVID-19 have shown widespread thrombosis with microangiopathy in histologic analysis.^7^ The importance of the vaso-occlusive disease mechanism in COVID-19 has been hypothesised.^8^

Perfusion was better preserved in areas of COVID-19 induced lung inflammation with a reduced/absent ventilation. Mathematical modeling of ventilation and perfusion in COVID-19 suggest that this reversed V/P mismatch (shunt perfusion) does not alone explain severe hypoxemia in COVID-19.^9,10^ The diversion of perfusion in V/P SPECT (dead space ventilation) appears to be a clinically important finding because ARDS from COVID-19 is characterised by elevated ventilation/perfusion mismatch with larger prevalence of ventilated, non-perfused lung units (dead space ventilation) contrary to perfused non-ventilated areas (shunt perfusion).^11^

All our COVID-19 patients showed signs of bilateral pulmonary inflammation affecting more the right lung. Simultaneous V/P SPECT and CT images in patients 3 and 4 indicate that the structural/morphological lesions in the chest CTs may be less pronounced than the functional defects in the patients’ lungs in COVID-19. Patients with inflammatory symptoms may not show pathological changes in radiological imaging modalities in early phases of COVID-19. Morphological changes may need a longer time to develop compared to functional defects which appear simultaneously with the subjective symptoms.^12^

In addition, other factors may worsen lung function in COVID-19 and contribute to dyspnoea, hypoxia and retention of CO2. Patient 4 showed a very rapid progression of COVID-19 symptoms and cardiac symptoms and V/P SPECT manifested perfusion pattern of cardiac congestion. V/P SPECT may signify cardiac damage by COVID-19 or show exacerbation of previous heart failure in COVID-19. In line with our previous experience, the dramatic fall of TPLF is associated with disturbance of blood gas homeostasis (hypoxia and hypercapnia) as in patient 4.

The case series clearly shows, that both ventilation and perfusion images are mandatory to interpret lung function in COVID-19 patients. In addition to PE diagnosis, V/P SPECT discloses other pathophysiological processes caused by SARS-CoV-2 virus in the lung that affect distribution of ventilation and perfusion. V/P mismatch and reversed mismatch quantified in percentage of the total lung volume may be extensive enough to diminish dramatically preserved matching ventilation/perfusion (ie, total preserved lung function or TPLF) and to induce severe hypoxemia in COVID-19.
